# Antioxidant/Anti-Inflammatory Effects of Caloric Restriction in an Aged and Obese Rat Model: The Role of Adiponectin

**DOI:** 10.3390/biomedicines8120532

**Published:** 2020-11-25

**Authors:** Daniele La Russa, Alessandro Marrone, Maurizio Mandalà, Rachele Macirella, Daniela Pellegrino

**Affiliations:** 1 Department of Pharmacy, Health and Nutritional Sciences, University of Calabria, 87036 Rende, Italy; 2 LARSO (Analysis and Research on Oxidative Stress Laboratory), University of Calabria, 87036 Rende, Italy; alessandro.marrone@unical.it; 3 Department of Biology, Ecology and Earth Sciences, University of Calabria, 87036 Rende, Italy; m.mandala@unical.it (M.M.); rachele.macirella@unical.it (R.M.)

**Keywords:** caloric restriction, inflammation, oxidative balance, adiponectin, plasma, white adipose tissue

## Abstract

Caloric restriction (CR) represents a powerful intervention for extending healthspan and lifespan in several animal models, from yeast to primates. Additionally, in humans, CR has been found to induce cardiometabolic adaptations associated with improved health. In this study, we evaluated in an aged and obese rat model the effect of long-term (6 months) caloric restriction (−40%) on the oxidative/inflammatory balance in order to investigate the underlining mechanisms. In plasma, we analyzed the oxidative balance by photometric tests and the adiponectin/tumor necrosis factor-α-induced gene/protein 6 (TSG-6) levels by Western blot analysis. In the white adipose tissue, we examined the protein levels of AdipoR1, pAMPK, NFκB, NRF-2, and glutathione S-tranferase P1 by Western blot analysis. Our results clearly showed that caloric restriction significantly improves the plasmatic oxidative/inflammatory balance in parallel with a major increase in circulating adiponectin levels. Additionally, at the level of adipose tissue, we found a positive modulation of both anti-inflammatory and antioxidant pathways. These adaptations, induced by caloric restriction, with the achievement of normal weight, suggest that inflammatory and redox imbalance in obese aged rats appear to be more linked to obesity than to aging.

## 1. Introduction

Obesity prevalence is constantly growing worldwide in all age groups and represents a serious publichealth problem, given its close correlation with several chronic diseases [1,2]. Obesity is not always the result of an excessive intake of food and/or lack of physical activity, but it is also favored by other factors (stress, drugs, or hormonal metabolic alterations) that alter the physiological mechanisms capable of regulating the supply of energy in relation to consumption, as happens, for example, with advancing age. In particular, the age-related changes in body fat distribution (increase in abdominal obesity) and metabolism (insulin resistance and metabolic syndrome) represent key factors in a vicious cycle that can accelerate the aging process itself and the progression of age-related diseases [3,4]. Lines of evidence from several studies have shown that obesity adversely affects health status and life span through cellular processes in a manner similar to aging [3,4].

Aging is a complex, multifactorial process driven by both intrinsic (genetic) and extrinsic (environmental) factors. The aging process is linked to homeostasis deterioration characterized by an alteration of both oxidative and inflammatory states. The free radical theory of aging has been proposed since 1956 and highlights how the accumulation of oxidized biomolecules during chronic oxidative stress is the basis of the age-related structural and functional alterations of all types of cells [5,6]. Over the past decades, the close link between free radicals and aging has found corroboration in several studies [7,8,9], although some published papers appear to contradict this theory [10,11]. The aging process is also characterized by a chronic low-grade of inflammation, defined as inflamm-aging [12], with an upregulation of proinflammatory cytokines and inflammatory compounds, all of which have been shown to be involved in the pathogenesis of age-related diseases [13]. The theory of oxidation and inflammation of aging, termed oxi-inflamm-aging, was proposed to better define what occurs during the aging process [14,15].

Additionally, obesity is characterized by a redox/inflammatory imbalance that is probably due to the endocrine activity of adipose tissue via the production of several adipokines involved in the development of inflammation, oxidative stress, abnormal lipid metabolism, increased production of insulin, and insulin resistance [16,17,18,19,20,21]. In particular, visceral obesity is associated with the development of chronic metabolic diseases through a convergence of a chronic inflammatory state and enhanced production of reactive oxygen species [21]. Lines of evidence from several studies have shown that obesity adversely affects health status and life span through cellular processes in a manner similar to aging, and the link between these processes appears to be adiponectin [3,4]. This adipokine, mainly secreted from adipocytes, modulates a number of metabolic processes and represents a key molecule in maintaining the functionality of many organs [22]; it is also involved in the development and progression of several obesity-related malignancies such as breast cancer [23,24]. As adiponectin physiologically decreases with age [25] and obesity negatively affects the decline in adiponectin levels [26], it can be assumed that obesity alters the aging process through the regulation of adiponectin.

Caloric restriction (CR) is a dietary intervention with a chronic reduction of total calorie intake without incurring malnutrition. CR activates a complex series of events and represents a powerful intervention for extending healthspan and lifespan in several animal models, from yeast to primates [27]. Although the detailed mechanisms remain to be established, several sets of experimental data have confirmed that CR induces metabolic remodeling in several organs and tissues, including white adipose tissue (WAT). In particular, CR modulates the adipokine expression profile in rodent WAT [28,29]. WAT plays a central role in the regulation of both energy storage and expenditure and is also directly involved in the regulation of lifespan. The current knowledge on the molecular and cellular events associated with CR underline how the beneficial effects of this dietary intervention can be due to an improvement in the plasma and tissue redox inflammatory profile [30,31,32].

Given the close relationship between obesity and aging/age-related diseases, the present studyundertakes to determine the similarities in underlying mechanisms related to both obesity and aging, focusing our attention on the key role played by adiponectin. Since the use of old animals is very important to phenocopy the systemic aging context, we used an elderly and obese rat model, a useful model that mimics the weight gain due to aging that occurs in humans.

## 2. Materials and Methods

### 2.1. Animals

Experiments were performed on young (13–15 weeks old, *n* = 6) and aged (72 weeks old, *n* = 12) male Sprague–Dawley rats. Animals were housed in the animal care facility of the University of Calabria (Italy) in a 12:12 h light–dark cycle and temperature-controlled rooms (22 °C) and had free access to food and water. The old animals were then divided into two subgroups: (1) Control rats were continued on an ad libitum diet of a standard laboratory chow (ssniff diet V1535, German; metabolizable energy 3.057 Kcal/Kg), while (2) food-restriction rats were fed a diet of the same chow, restricted to 60% of the intake measured by weight in pairedcontrol chow-fed rats. The food restriction diet was carried on for a total period of 6 months, and thenthe aged animals (control and treated) were sacrificed at 24 months old. Water and food intakes were recorded every other day, while body mass was recorded monthly. Animals were euthanized with isoflurane (4%) followed by cervical transection, and several tissueswere immediately removed: abdominal fat, kidney, blood, and skeletal muscle. All experiments were carried out in accordance with the European Guidelines for the care and use of laboratory animals (Directive 26/2014/EU) and were approved by the local ethical committee of the University of Calabria and by the Italian Ministry of Health (license n.295/2016-PR).

### 2.2. Measurement of Plasma Oxidative Status

Plasma oxidative status was evaluated through photometric measurement kits combined with a free radical analyzer system and a spectrophotometric device reader (FREE Carpe Diem, Diacron International, Grosseto, Italy), which are routinely used in our laboratory in both human and rat models [33,34,35,36,37,38]. The diacron-reactive oxygen metabolite (dROM) test was employed for analyzing the total amount of hydroperoxides in plasma samples. The normal range of the test results was 250–300 U.CARR (Carratelli Units), where 1 U.CARR corresponds to 0.8 mg/L of hydrogen peroxide. Total plasma antioxidant capacity was measured using a biological antioxidant capacity (BAP) test. Results are expressed in µmol/L of the reduced ferric ions.

### 2.3. Western Blot and Densitometric Analysis

White adipose tissue samples were lysed in ice-cold RIPA buffer containing a protease inhibitor cocktail (Sigma-Aldrich, Milan, Italy) and centrifuged for 20 min at 20,817× *g* at 4 °C. After removing the layer of lipids, supernatants were collected and the protein concentration was quantified using the Bradford method (Sigma, St Louis, MO, USA). For blood samples, after centrifuging for 15 min at 1500× *g* at 4 °C, plasma was collected and diluted (1:10 *v/v*) in ice-cold RIPA buffer containing a protease inhibitor cocktail (Sigma-Aldrich, Milan, Italy). The same amounts of total protein lysate were heated for 5 min in Laemmli buffer (SigmaAldrich, Milan, Italy), separated by sodium dodecyl sulfate polyacrylamide gel electrophoresis (SDSPAGE) in a Bio-Rad Mini Protean III machine and then electroblotted onto a nitrocellulose membrane (NitroBind, Maine Manufacturing, Maine, USA) using a mini transblot (BioRad Laboratories, Hercules, CA, USA). The gels for immunoblot analyses were transferred to a nitrocellulose membrane and incubated overnight at 4 °C, with primary antibodies diluted in TBS-T directed against AdipoR1, pAMPK, NFκB, NRF-2, GSTP1, TSG6, and adiponectin, followed by species-specific peroxidase-linked secondary antibodies (1:2000; Santa Cruz Biotechnology Inc., Dallas, TX, USA) for 1 h at room temperature. Immunodetection of protein bands was performed with an enhanced chemiluminescence kit (Western Blotting Luminol Reagent, Santa Cruz Biotechnology Inc.), and the membranes were exposed to X-ray films (Ultracruz Autoradiography Film, Santa Cruz Biotechnology Inc.). The films were then scanned, and densitometric analysis of the bands was performed using ImageJ software (1.52a version, National Institutes of Health, Bethesda, MD, USA). Beta actin and serum albumin were used as loading controls, respectively, for tissue and blood protein normalization.

### 2.4. Statistical Analysis

Data were analysed by one-way analysis of variance (ANOVA), followed by the Bonferroni multiple comparisons test using GraphPad/Prism version 5.01 statistical software (SAS Institute, Abacus Concept Inc., Berkeley, CA, USA. All the results are expressed as the mean ± SE.

## 3. Result

### 3.1. Effects of CR on Obesity and Plasmatic Oxidative Balance

**Figure 1:**
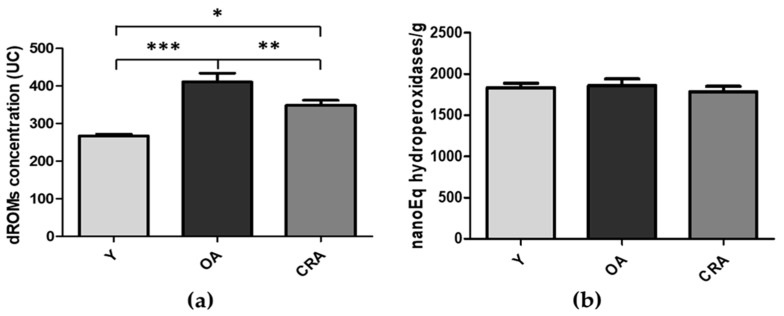
Plasmatic values of oxidative stress (d-ROMs) (**a**) and antioxidant capacity efficiency (BAP) (**b**) tests in young rats (Y), obese aged (OA), and aged undergoing calorie restriction (CRA)rats. Data are means ± SE of five determinations for each animal (*n* = 6). Statistical differences were evaluated by one-way ANOVA, followed by Bonferroni’s multiple comparison test (* *p* < 0.05; ** *p* < 0.001; *** *p* < 0.0001).

In laboratory rodents, obesity is defined as the achievement of a 20% increase in body mass index [39]. In our obesity model, the aged rats (2 years old) showed a significant increase in body weight, greater than 45%, compared to young rats (13–15 weeks old). Furthermore, these aged specimens had a significant plasma oxidative imbalance, with a considerable increase in oxidative stress and a parallel and severe decrease in the antioxidant barrier. In our model, the calorie restriction (−40%) for 6 months determined a significant decrease in body weight (−34%) vs. control and, at the same time, significantly improved the oxidative balance with a remarkable decrease in oxidative stress, independent of changes in the plasmatic antioxidant barrier. The body weight data and the trend of both oxidative stress (d-ROMs) and antioxidant capacity efficiency (BAP) in plasma of young rats (Y), obese aged (OA), and aged undergoing calorie restriction (CRA) rats are reported in Figure 1.

### 3.2. Effects of CR on Plasmatic Adiponectin Levels and Inflammation Markers

**Figure 2:**
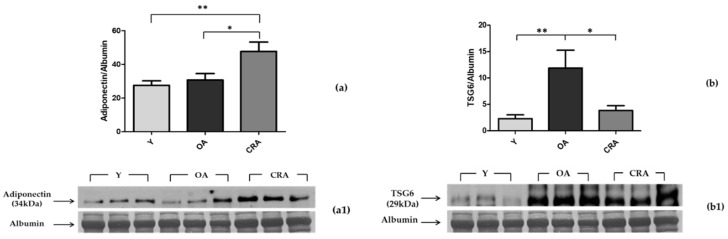
Western blotting of adiponectin (**a**) and TSG6 (**b**) in plasma samples of Y, OA, and CRA rats; (**a1**,**b1**) show the densitometric quantification of the blots. Protein loading was verified by plasmatic albumin level. Data are means ± SE of five determinations for each animal (*n* = 6). Statistical differences were evaluated by one-way ANOVA, followed by Bonferroni’s multiple comparison test (* *p* < 0.05; ** *p* < 0.001).

To assess the possible role played by adiponectin in the antioxidant effect of CR, we evaluated its plasmatic protein levels through Western blotting analysis. In parallel, we also evaluated the plasmatic protein levels of TSG-6, a multifunctional protein associated with inflammation. Our results clearly indicate that the obesity condition associated with aging does change the circulating adiponectin levels and also induces a significant increase in TSG-6 levels, confirming the low-grade proinflammatory state found in both obesity and aging (Figure 2a). The caloric restriction determines an increase in the adiponectin plasma levels (doubled compared to both young and aged rats) and a significant reduction of the TSG-6 proinflammatory marker, which reverts to the values of the young rats (Figure 2b).

### 3.3. Effects of CR on AdipoR1–pAMPK–NFκB Pathway in Adipose Tissue

**Figure 3:**
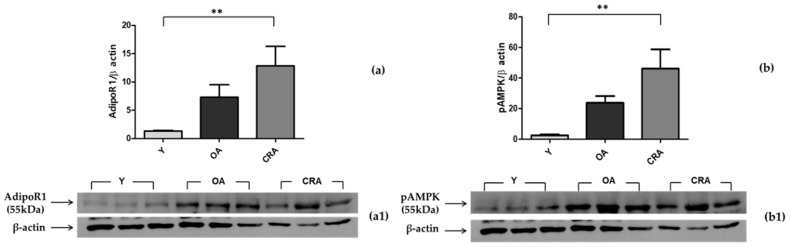
Western blotting of AdipoR1 (**a**) and AMPK activation (pAMPK) (**b**) in adipose tissue samples of Y, OA, and CRA rats; (**a1**,**b1**) show the densitometric quantification of the blots. Protein loading was verified by using the anti-β-actin antibody. Data are means ± SE of five determinations for each animal (*n* = 6). Statistical differences were evaluated by one-way ANOVA, followed by Bonferroni’s multiple comparison test (** *p* < 0.001).

**Figure 4:**
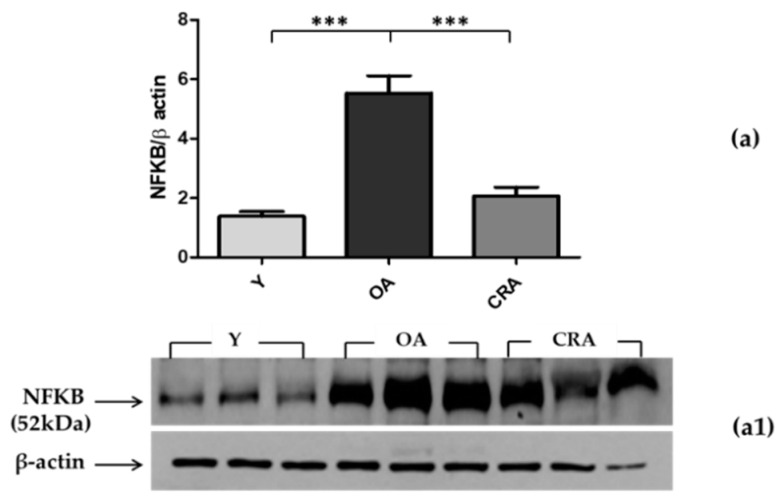
Western blotting of NFκB (**a**) in adipose tissue samples of Y, OA, and CRA rats; (**a1**) shows the densitometric quantification of the blots. Protein loading was verified by using the anti-β-actin antibody. Data are means ± SE of five determinations for each animal (*n* = 6). Statistical differences were evaluated by one-way ANOVA, followed by Bonferroni’s multiple comparison test (*** *p* < 0.0001).

Since adipose tissue represents a crucial junction in inflammatory processes and it is also a reserve of adipokines, we analyzed the AdipoR1–pAMPK–NFκB pathway at the level of adipose tissue. Through its receptor, adiponectin mediates AMPK activation (pAMPK), which is involved in the inhibition of NFκB activation and the suppression of inflammation. In our model, the obesity condition associated with aging determines an upregulation of AdipoR1, with a consequent increase of the AMPK active form in adipose tissue (Figure 3a,b). Despite the activation of this anti-inflammatory pathway, NFκB levels found in obese and aged subjects (control) were high compared to the young rats. CR, with the achievement of normal weight and the rise of both AdipoR1 and pAMPK, significantly inhibited NFκB expression (Figure 4) and consequently suppressed the proinflammatory state, which appears to be more linked to obesity than aging (Figure 2b).

### 3.4. Effects of CR on Antioxidant Enzymes in Adipose Tissue

**Figure 5:**
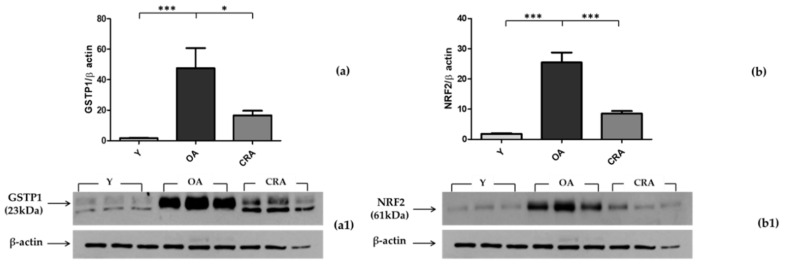
Western blotting of GSTP1(**a**) and NRF2(**b**) in adipose tissue samples of Y, OA, and CRA rats; (**a1**,**b1**) show the densitometric quantification of the blots. Protein loading was verified by using the anti-β-actin antibody. Data are means ± SE of five determinations for each animal (*n* = 6). Statistical differences were evaluated by one-way ANOVA, followed by Bonferroni’s multiple comparison test (* *p* < 0.05; *** *p* < 0.0001).

To analyze the oxidative imbalance linked to obesity detected at the plasma level, we evaluated two important cytoplasmatic antioxidant enzymes, SOD1 and GSTP1, in adipose tissue. SOD1, the most important preventive antioxidant, showed no significant changes in the three groups of rats (data not shown). Regarding the GSTP1 monomer (23 kDa), the form with antioxidant and proliferative activity, our results showed a significant increase in obese rats while it is reduced in CRArats (Figure 5a). We also evaluated NRF2, a transcription factor physiologically present in the cytosol that migrates to the nucleus only under stress conditions, where it stimulates the expression of antioxidant genes. Our results clearly showed that in obese aged rats, the cytoplasmic fraction of NRF2 is significantly increased, while in CRA rats, it is significantly decreased (Figure 5b).

## 4. Discussion

This study was designed to evaluate the effect of long-term (6 months) caloric restriction (−40%) on the oxidative/inflammatory balance in an aged and obese rat model and the putative role of adiponectin. Our results showed notable alterations in redox/inflammatory status in our aging obesity model compared to young rats and revealed that CR treatment improves the plasmatic and cellular capacity to neutralize oxidative insults and induces a significant reduction of proinflammatory markers. This improvement of the redox/inflammatory status during CR is connected with an important increase in adiponectin plasma levels, thus suggesting an important role of this adipokine in the antioxidant/anti-inflammatory properties of the organism. Additionally, at the level of adipose tissue, we found a positive modulation of both anti-inflammatory and antioxidant pathways.

During the aging process, there is often a gradual increase in body weight and, typically, the fat is redistributed from the subcutaneous to the abdominal deposits and the liver, and this phenotypic change can affect energy metabolism and systemic insulin resistance [40]. Aged rodents develop increased fat mass, with close similarities to aged humans [41], making these animals excellent experimental models for analyzing both obesity and aging. In our experimental model, age-related physiological weight gain leads to two-year-old rats with 45% increased body weight compared to the young animals and, therefore, with overt obesity [39]. These aged and obese specimens, according to the oxidation–inflammation theory of aging [14,15], showed a considerable increase in oxidative stress, a parallel and severe decrease in the antioxidant barrier, and a significant upregulation of TSG-6, a multifunctional protein associated with inflammation [42]. Our results clearly indicate that the obesity condition associated with aging induces oxidative/inflammatory stress situations, confirming the oxidative imbalance and low-grade proinflammatory state found in both obesity and aging [4]. In our aged and obese rat model, we did not detect changes in plasma adiponectin levels with respect to the young animals. Several factors may influence adiponectin levels and activity. In obesity, as the metabolic role of adipocytes changes, the secretion of adiponectin decreases in both humans and rodents, and this is associated with chronic inflammation [4,26,43]. Data on adiponectin levels and aging have revealed conflicting results; some authors have indicated that plasmatic adiponectin levels increase with advancing age [44], while others have reported no age-related changes in its levels [4,25].

Interestingly, in our model, the long-term caloric restriction reversed obesity, determining a significant decrease in body weight (−34%) and, in parallel, increasing the adiponectin plasma levels, which were doubled compared to both young and obese aged control rats. The increased adiponectin plasma levels led to the improvement of the proinflammatory state, with a severe reduction of the TSG-6 proinflammatory marker, which reverted to the values of the young rats. The protein TSG6, a critical anti-inflammatory cytokine detected in the context of many inflammatory diseases, is produced in response to inflammatory mediators, mostly after TNF-α stimulation [42]. The anti-inflammatory and cytoprotective effects of adiponectin have long been known [45,46,47], and caloric restriction is also considered a possible strategy to better body metabolic and inflammatory profiles [48,49]. The role played by adiponectin and caloric restriction in maintaining/restoring the oxidative balance is less clear. A wide number of studies have reported a negative correlation of adiponectin levels with markers of oxidative stress [50] and a cause–effect relationship between the metabolic syndrome and a deteriorated oxidative status related to the altered secretion of adipokines [51]. In addition, it has been suggested that oxidative stress can inhibit adiponectin expression in obesity, but the mechanism underlying this regulation is unclear [19]. Our results show a clear improvement in the plasma redox status with a remarkable decrease in oxidative stress, independent of changes in the plasmatic antioxidant barrier.

To investigate the mechanisms triggered by caloric restriction in the improvement of oxidative/inflammatory status in our model, we analyzed anti-inflammatory and antioxidant pathways at the level of adipose tissue, focusing on the key role played by adiponectin. Adipose tissue represents a crucial junction in inflammatory/oxidant processes, and it is also a reserve of adipokines. The biological activities of adiponectin are closely related to the activation of AMP-activated protein kinase (AMPK), a key enzyme in the regulation of cellular energy homeostasis and fatty acid metabolism [52,53]. Several authors have reported that AMPK plays an important role in the modulation of inflammation, and AMPK activators exert a protective effect in animal models of inflammatory diseases [45]. In addition, AMPK can inhibit ROS generation [53,54]. Recently, it has been suggested that adiponectin may control inflammation by upregulating AMPK phosphorylation and then reducing the ROS-initiated inflammatory response [54]. Moreover, in cellular models, adiponectin treatment linearly decreased the production of ROS [54]. Through its receptor, adiponectin exerts an anti-inflammatory response by AMPK activation (pAMPK), which is involved in the inhibition of NFκB activation and consequent TNF-α and IL-1β downregulation [45].

In our model, the obesity condition associated with aging determines an upregulation of AdipoR1, with a consequent increase of the AMPK active form in adipose tissue. Despite the activation of this anti-inflammatory pathway, the NFκB levels found were high compared to young rats. CR, with the achievement of normal weight, enhances the stimulation of both AdipoR1 and pAMPK, with significant inhibition of NFκB expression and, consequently, suppression of the proinflammatory state.

To better understand the tissue redox balance, we also focused on the expression of two important cytoplasmatic antioxidant enzymes, SOD1 and GSTP1, in adipose tissue. CR has been shown to positively affect tissue redox homeostasis by the enhancement of endogenous antioxidant systems [55]. In our model, the expression of SOD1, the most important preventive antioxidant, showed no significant changes in all groups of rats used, while the GSTP1 monomer, the form with antioxidant and proliferative activity, showed a negative modulation by CR treatment. We also evaluated the expression of NRF2, a transcription factor that is physiologically present in the cytosol that migrates to the nucleus only under stress conditions, where it stimulates the expression of antioxidant genes. Our results clearly show that in obese aged rats, the cytoplasmic fraction of NRF2 is significantly increased and that CR induces a substantial decrease, probably due to a translocation effect in the nucleus to modulate the expression of antioxidant genes.

Overall, our results highlight that the long-term caloric restriction leads to the achievement of normal weight and induces a number of adaptations that improve the redox/inflammatory status at both plasma and tissue levels, suggesting that inflammatory and redox imbalances in obese aged rats appear to be more linked to obesity than to aging.
